# Lipid fingerprinting by MALDI Biotyper Sirius instrument fails to differentiate the three subspecies of the *Mycobacterium abscessus* complex

**DOI:** 10.1128/jcm.01484-24

**Published:** 2025-03-14

**Authors:** Mitsunori Yoshida, Hanako Fukano, Koji Yahara, Satoshi Nakano, Takeshi Komine, Masato Suzuki, Azumi Fujinaga, Kohei Doke, Yoshihiko Hoshino

**Affiliations:** 1 Department of Mycobacteriology, Leprosy Research Center, National Institute of Infectious Diseases 231182 , Higashi-Murayama, Tokyo, Japan; 2 Antimicrobial Resistance (AMR) Research Center, National Institute of Infectious Diseases 13511 , Higashi-Murayama, Tokyo, Japan; 3 Bruker JAPAN K.K, Kanagawa, Japan; University of Manitoba, Winnipeg, Manitoba, Canada

**Keywords:** lipid profiling, mass spectrometry, ATS Clinical Guideline, machine learning, clinical isolate

## LETTER

The number of patients with *Mycobacterium abscessus* complex (MABC) pulmonary diseases is steadily increasing. MABC consists of three subspecies and has been described as an “antibiotic nightmare” due to its resistance to most antibiotics. The 2020 ATS Clinical Practice Guideline recommends distinguishing MABC subspecies due to their differing macrolide susceptibilities (1). Unfortunately, methods such as high-performance liquid chromatography (HPLC), multilocus sequence typing (MLST), and whole-genome sequencing (WGS) are inefficient due to high cost, complexity, and time requirements. Matrix-assisted laser desorption ionization-time of flight (MALDI-TOF) mass spectrometry (MS) is widely used in laboratories as a cost-effective and accurate method for identifying mycobacterial species. However, distinguishing MABC subspecies using proteomic profiling remains challenging (2–4).

The third-generation Bruker MALDI Biotyper (MBT) Sirius has enhanced capabilities, including the detection of phospholipids and glycolipids using negative ion mode. Mycobacterial cell walls are lipid-rich, and if lipid structure diversity serves as a species-specific fingerprint, this method may provide an alternative for microbial identification. A recent study suggested that lipid profiling could differentiate MABC subspecies, but its accuracy has not been fully assessed (5). This study aimed to evaluate the accuracy of differentiating the three MABC subspecies using lipid profiling on a set of MABC isolates.

We analyzed 149 clinical or environmental isolates (listed in Table S1) that had undergone WGS (6). Mass spectra were acquired using the MBT Sirius in negative ion mode, and data analysis was conducted using ClinProTools 3.0 (Bruker). A discrimination model was developed based on a set of clinical isolates and three reference strains representing the MABC phylogenetic tree (Fig. S1 and S2). This model was then applied to the remaining isolates. Additionally, machine learning models—including random forest, neural networks, and support vector machine—were employed using the MSclassifR package (v0.3.2, https://github.com/agodmer/MSclassifR_examples).

**Fig 1:**
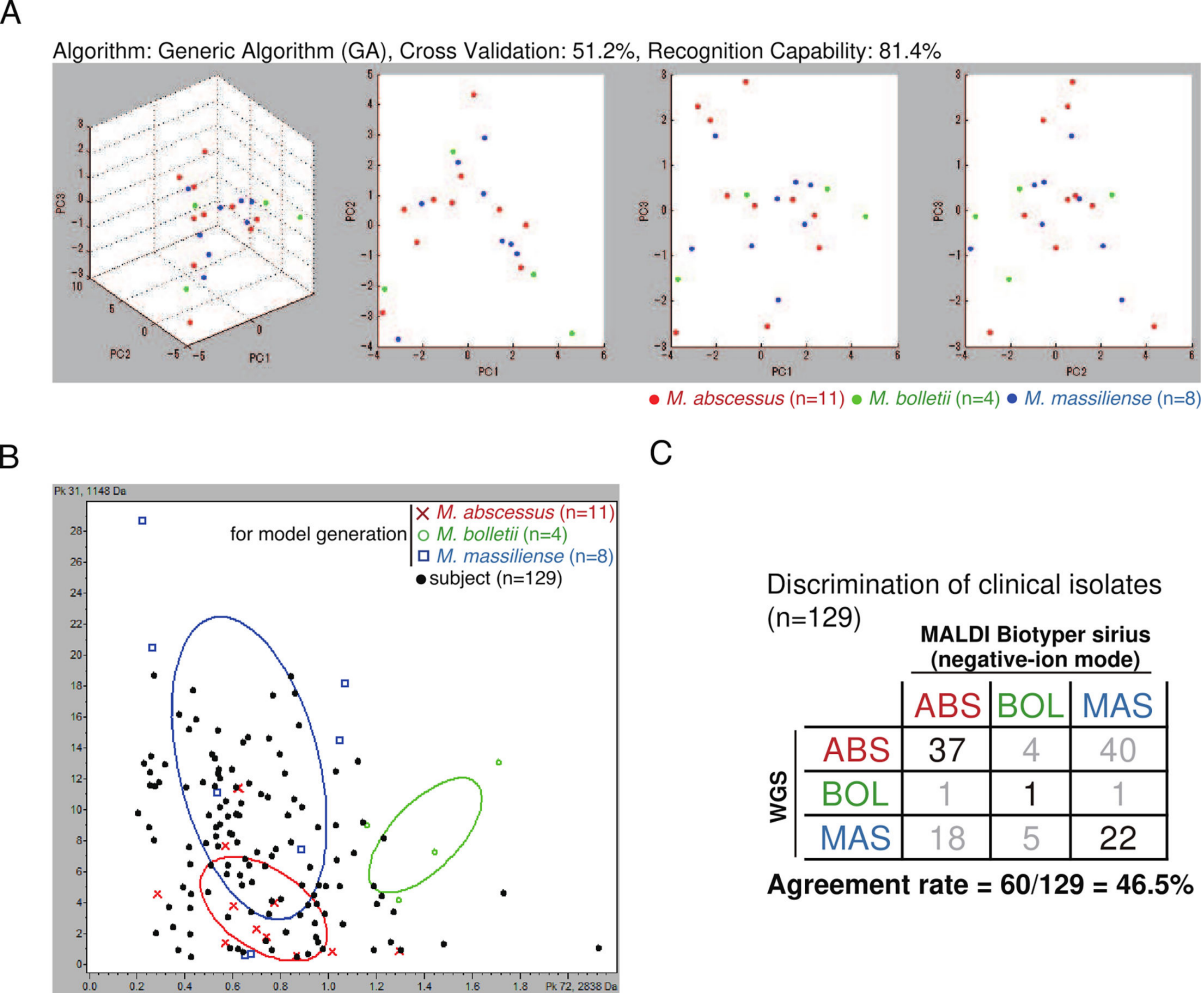
(**A**) Principal component analysis (PCA) of the peaks (760–3200 m/z) of *M. abscessus* complex. A scatter plot of the first three principal components (PC1 to PC3) of the peak lists of MBT Sirius with negative-ion mode shows the distribution of the isolates of *M. abscessus* subsp. *abscessus* (*M. abscessus*, red, *n* = 11), *M. abscessus* subsp. *massiliense* (*M. massiliense*, blue, *n* = 8), and *M. abscessus* subsp. *bolletii* (*M. bolletii*, green, *n* = 4). These 23 isolates, including three reference strains (ATCC 19977, JCM 15300, and BD), cover the genomic diversity of the MABC (arrows in Fig. S1). (**B**) A scatter plot of the two peaks of MBT Sirius with negative-ion mode that are statistically most significant peaks to identify MABC subspecies (peaks 72 and 31) are shown. A set of MABC isolates (black, *n* = 129) were coplotted onto the two-dimensional space between these peaks. (**C**) Agreement rate between subspecies identification using WGS data and MBT Sirius data. Classification of isolates (*n* = 129) was performed using a model generated with ClinProTools 3.0 (GA, same model as A). Subspecies identification using WGS data was performed as described previously (6).

Our models failed to differentiate the three subspecies, which overlapped on principal component planes (Fig. 1A; Fig. S2). Even in the two-dimensional space of the most statistically significant peaks (peaks 72 and 31), *M. abscessus* subsp. *abscessus* and *Mycobacterium abscessus* subsp*.massiliense* could not be separated (Fig. 1B). The concordance rate between lipid fingerprint-based and WGS-based identification was only 56% (Fig. 1C; Fig. S2). Even with advanced machine learning algorithms, classification accuracy remained at 50% (Table S2). Khor et al. used ethanol preparation and positive ion mode for MS analysis, concluding that wave patterns between 1,200 and 1,450 m/z could differentiate the subspecies. However, when negative ion mode was used, subspecies-specific peaks in this range were not observed (Fig. S3) (5).

Our findings indicate that lipid fingerprinting alone is insufficient for differentiating MABC subspecies. A recent study successfully used machine learning and proteomic profiling in positive ion mode, but this method has not yet been incorporated into Bruker’s commercial software and will take time to implement clinically (7). Additionally, lipid profiling data did not improve with machine learning. Thus, alternative methods, such as DNA chromatography (6) or Genotype NTM-DR (8), are required for accurate subspecies differentiation in clinical practice. Further advancements in MALDI-TOF MS-based methods are necessary for routine clinical use.

## SUPPLEMENTAL MATERIAL

Supplemental methods and figures jcm.01484-24-s0001.docx Supplemental methods and Figures S1 to S3.

Supplemental tables jcm.01484-24-s0002.xlsx Tables S1 and S2.

ASM does not own the copyrights to Supplemental Material that may be linked to, or accessed through, an article. The authors have granted ASM a non-exclusive, world-wide license to publish the Supplemental Material files. Please contact the corresponding author directly for reuse.

## References

[1] Daley CL, Iaccarino JM, Lange C, Cambau E, Wallace RJ Jr, Andrejak C, Böttger EC, Brozek J, Griffith DE, Guglielmetti L, Huitt GA, Knight SL, Leitman P, Marras TK, Olivier KN, Santin M, Stout JE, Tortoli E, van Ingen J, Wagner D, Winthrop KL. 2020. Treatment of nontuberculous mycobacterial pulmonary disease: an official ATS/ERS/ESCMID/IDSA clinical practice guideline: executive summary. Clin Infect Dis 71:e1–e36. doi:10.1093/cid/ciaa241 32628747 PMC7768748

[2] Brown-Elliott BA, Fritsche TR, Olson BJ, Vasireddy S, Vasireddy R, Iakhiaeva E, Alame D, Wallace RJ, Branda JA. 2019. Comparison of two commercial matrix-assisted laser desorption/ionization-time of flight mass spectrometry (MALDI-TOF MS) systems for identification of nontuberculous mycobacteria. Am J Clin Pathol 152:527–536. doi:10.1093/ajcp/aqz073 31314059 PMC6733354

[3] Luo L, Liu W, Li B, Li M, Huang D, Jing L, Chen H, Yang J, Yue J, Wang F, Chu H, Zhang Z. 2016. Evaluation of matrix-assisted laser desorption ionization-time of flight mass spectrometry for identification of Mycobacterium abscessus subspecies according to whole-genome sequencing. J Clin Microbiol 54:2982–2989. doi:10.1128/JCM.01151-16 27682129 PMC5121389

[4] Fangous MS, Mougari F, Gouriou S, Calvez E, Raskine L, Cambau E, Payan C, Héry-Arnaud G. 2014. Classification algorithm for subspecies identification within the Mycobacterium abscessus species, based on matrix-assisted laser desorption ionization-time of flight mass spectrometry. J Clin Microbiol 52:3362–3369. doi:10.1128/JCM.00788-14 25009048 PMC4313163

[5] Jia Khor M, Broda A, Kostrzewa M, Drobniewski F, Larrouy-Maumus G. 2021. An improved method for rapid detection of Mycobacterium abscessus complex based on species-specific lipid fingerprint by routine MALDI-TOF. Front Chem 9:715890. doi:10.3389/fchem.2021.715890 34386482 PMC8353234

[6] Yoshida M, Sano S, Chien J-Y, Fukano H, Suzuki M, Asakura T, Morimoto K, Murase Y, Miyamoto S, Kurashima A, Hasegawa N, Hsueh P-R, Mitarai S, Ato M, Hoshino Y. 2021. A novel DNA chromatography method to discriminate Mycobacterium abscessus subspecies and macrolide susceptibility. EBioMedicine 64:103187. doi:10.1016/j.ebiom.2020.103187 33446475 PMC7910664

[7] Rodríguez-Temporal D, Herrera L, Alcaide F, Domingo D, Héry-Arnaud G, van Ingen J, Van den Bossche A, Ingebretsen A, Beauruelle C, Terschlüsen E, Boarbi S, Vila N, Arroyo MJ, Méndez G, Muñoz P, Mancera L, Ruiz-Serrano MJ, Rodríguez-Sánchez B. 2023. Identification of Mycobacterium abscessus subspecies by MALDI-TOF mass spectrometry and machine learning. J Clin Microbiol 61:e0111022. doi:10.1128/jcm.01110-22 36602341 PMC9879094

[8] Kehrmann J, Kurt N, Rueger K, Bange FC, Buer J. 2016. GenoType NTM-DR for identifying Mycobacterium abscessus subspecies and determining molecular resistance. J Clin Microbiol 54:1653–1655. doi:10.1128/JCM.00147-16 27030487 PMC4879307

