# Supplementary material for: Lipid fingerprinting by MALDI Biotyper Sirius instrument fails to differentiate the three subspecies of the Mycobacterium abscessus complex
Source: J Clin Microbiol. 2025 Mar 14;63(4):e01484-24. doi: 10.1128/jcm.01484-24 (PMC11980375; doi:10.1128/jcm.01484-24)
Supplement: Supplemental methods and figures — S1 to S3. [file jcm.01484-24-s0001.docx]

***Supplemental material***

**Lipid Fingerprinting by MALDI Biotyper Sirius Instrument Fails to Differentiate the Three Subspecies of the Mycobacterium abscessus Complex**

Mitsunori Yoshida, Hanako Fukano, Koji Yahara, Satoshi Nakano, Takeshi Komine, Masato Suzuki, Azumi Fujinaga, Kohei Doke, Yoshihiko Hoshino

**Supplemental Methods**

**Bacterial isolates**

A total of 149 clinical or environmental MABC isolates were used in this study (Table S1). All isolates were identified by whole-genome sequencing as previously described (1). *M. abscessus* JCM 13569 (=ATCC 19977), *M. massiliense* JCM 15300 and *M. bolletii* JCM 15297 (=BD) type strains were obtained from the Japan Collection of Microorganisms of the Riken Bio-Resource Center (BRC-JCM; Ibaraki, Japan). All bacterial strains/isolates were subcultured on 7H10 agar plates supplemented with 10% OADC for up to 7 to 10 days.

**Sample preparation and spectra acquisition using MBT Sirius**

Total mycobacterial lipids were extracted as described elsewhere with some modifications (2). In brief, according to the manufacturer's instructions, a loopful (1 μm) of biomass was processed by the MBT Lipid Xtract kit (Bruker, Germany). Spectra of the lipid profiles were obtained using a MALDI Biotyper Sirius system (Bruker, Germany) in negative ion mode with mass-to-charge ratios (*m/z*) in the range of 760 to 3,200 using flexControl software v3.4.

**Data processing and analysis**

The spectra were analyzed as described previously (2). Briefly, peak spectra were detected based on the *t*-test, analysis of variance (ANOVA), and the Wilcoxon or Kruskal–Wallis test in ClinProTools v3.0 with default settings (Bruker, Germany). Using spectra of a set of representative clinical isolates and the three reference strains (arrows in Fig. S1 and Fig. S2), predictive models were generated by three algorithms, including the genetic algorithm (GA), supervised neural network (SNN), and quick classifier (QC) implemented in ClinProTools v3.0 (3). As described elsewhere (4), we also carried out machine-learning (ML) based signal processing using the MSclassifR and the MALDIquant packages (5, 6). Briefly, informative peaks of spectra were detected by following steps: (I) intensity transformation, (II) spectrum smoothing, (III) baseline processing, (IV) intensity calibration, (V) spectrum alignment (Godmer et al., 2024) using spectrum obtained from 147 clinical isolates and the three reference strains. The dataset is split into training (70%) and test (30%) datasets for the machine-learning step. Three algorithms implemented in MSclassifR, including the recursive feature elimination algorithm coupled with random forests (RFE-RF), RFE coupled with logistic regression (RFE-Glmnet) and sparse partial least squares discriminant analysis (sPLSDA), were used for peak detection. A Leave-One-Out Cross-Validation (LOOCV) with 5 to 8 peaks was used for multiple steps of the peak detection, including the thresholding step, interpretation step, and prediction step. The LogReg function in MSclassifR package was used to generate ML-based prediction models with k-fold cross-validation (k = 2, and 2 repeats), including linear or nonlinear with neural networks (nnet), random forests (rf), support vector machines with linear kernel (svm), or eXtreme Gradient Boosting (xgb). Accuracy was defined as; (True Positive + True Negative)/(True Positive + False Positive + True Negative + False Negative).

**Supplementally figure legends**

**Figure S1**

Maximum likelihood core-gene phylogeny of MABC isolates. Core gene alignment of 149 isolates and three reference strains (*M. abscessus* subsp. *abscessus* ATCC19977, *M. abscessus* subsp. *massiliense* JCM 15300, and *M. abscessus* subsp. *bolletii* BD) of MABC was generated as described previously (6). The scale bar indicates the mean number of nucleotide substitutions per site (Snps/Site) on the respective branch. Samples are highlighted based on inclusion in three major clusters corresponding to MABC subspecies. Arrowheads indicate MABC isolates used for generating classification models. Mass spectra were acquired using MBT sirius with negative ion mode of MABC isolates and reference strains.

**Figure S2**

Examples of discrimination models. (A), (B) Maximum likelihood core-gene phylogeny of MABC isolates are the same as Fig. S1. Arrowheads indicate MABC isolates used for generating classification models. Principal component analysis (PCA) of the peaks (760-3200 m/z) of *M. abscessus* complex are shown. Agreement rate between subspecies identification using WGS data and MBT sirius data are indicated. Classification of isolates was performed using a model generated with ClinProTools 3.0.

**Figure S3**

Mass spectra of MABC reference strains and representative clinical isolates. (A) Comparison of two lipid extraction methods. Mass spectra of three reference strains (ATCC19977, JCM 15300, and BD) of MABC acquired by the MBT sirius system using the MBT Lipid Xtract kit or a previous method (Khor *et al.* 2021) are shown. (B) Mass spectra of three reference strains (ATCC19977, JCM 15300, and BD) and clinical isolates of MABC in the range of 1100 to 1600 *m/z* are shown.

**Supplemental Figures**

**Figure S1**

**
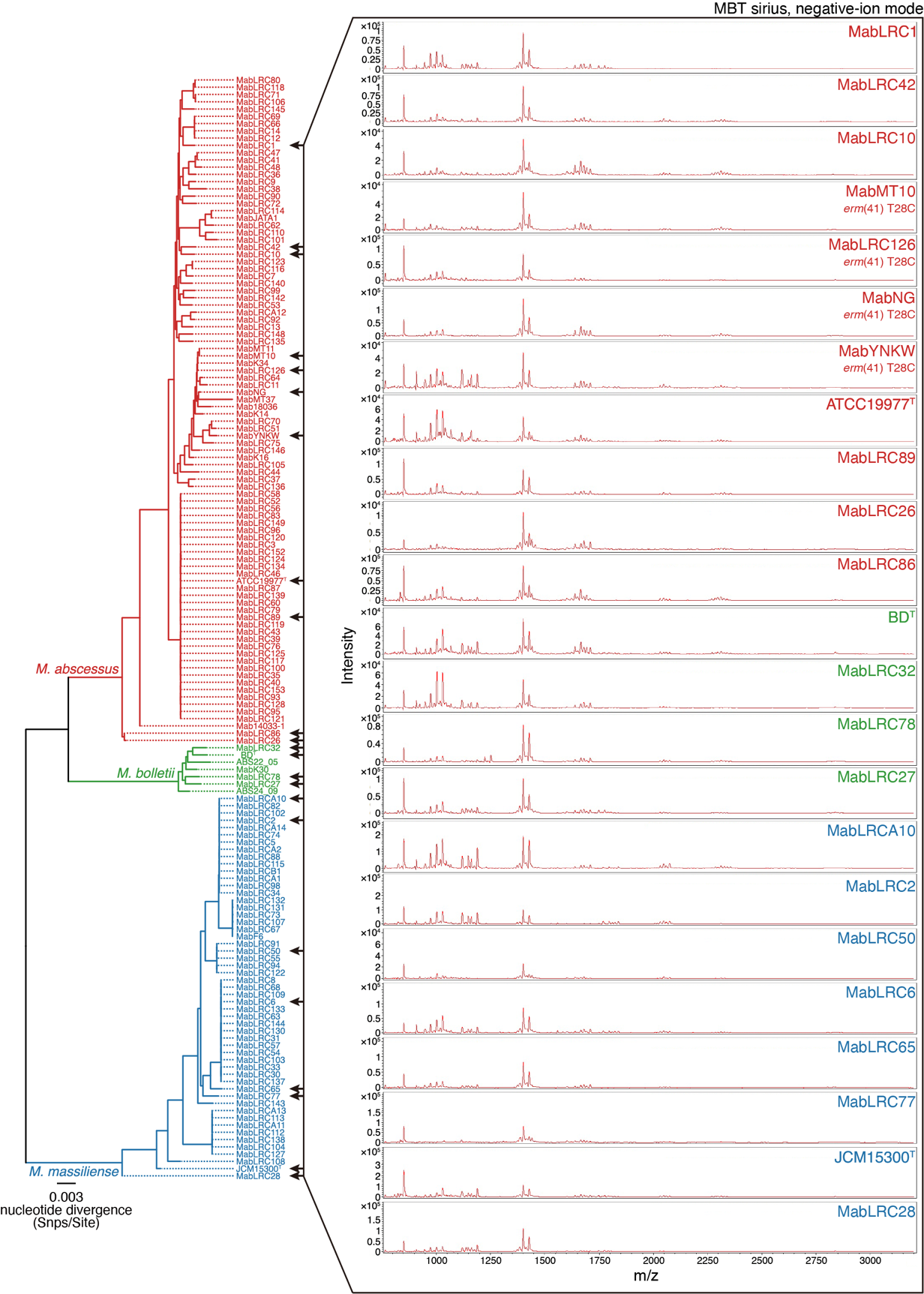
**

**Figure S2**

**
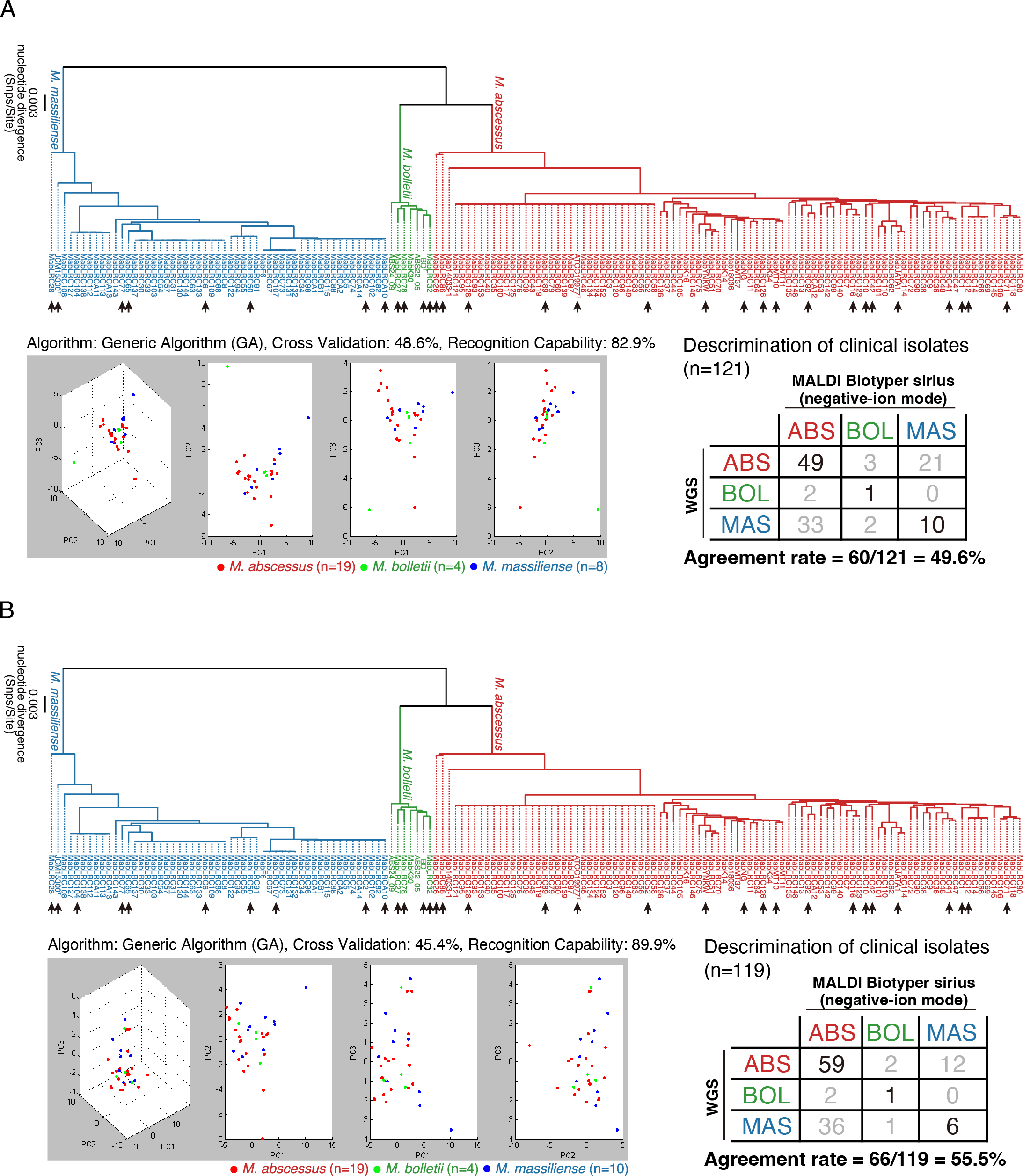
**

**Figure S3**

**
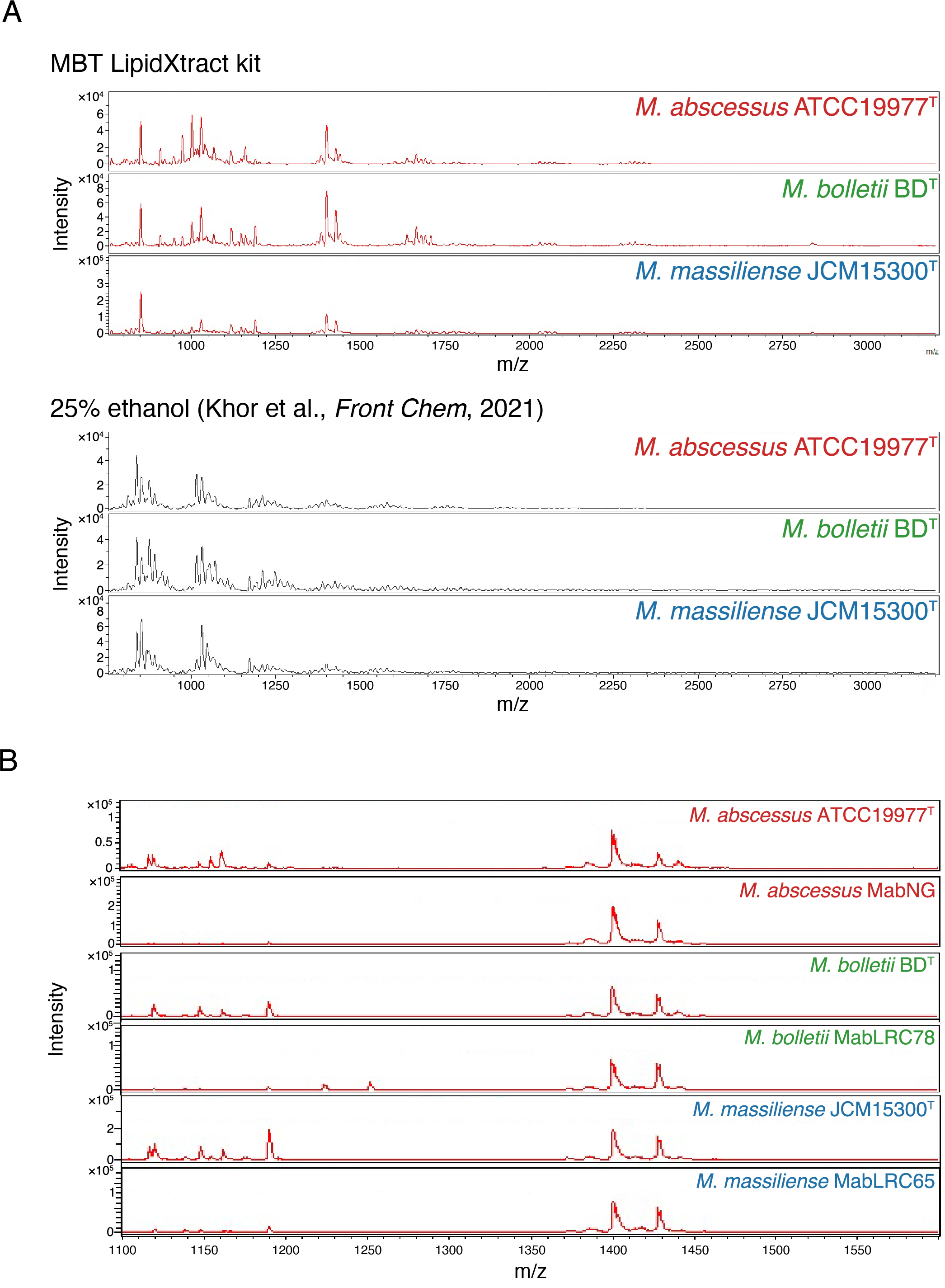
**

**Supplemental References**

1. Yoshida M, Sano S, Chien J-Y, Fukano H, Suzuki M, Asakura T, Morimoto K, Murase Y, Miyamoto S, Kurashima A, Hasegawa N, Hsueh P-R, Mitarai S, Ato M, Hoshino Y. 2021. A novel DNA chromatography method to discriminate Mycobacterium abscessus subspecies and macrolide susceptibility. EBioMedicine 103187.

2. Komine T, Fukano H, Yoshida M, Miyamoto Y, Nakaya M, Fujinaga A, Doke K, Hoshino Y. 2025. A rapid and simple MALDI-TOF MS lipid profiling method for differentiating Mycobacterium ulcerans from Mycobacterium marinum. J Clin Microbiol https://doi.org/10.1128/JCM.01400-24.

3. Weis C V., Jutzeler CR, Borgwardt K. 2020. Machine learning for microbial identification and antimicrobial susceptibility testing on MALDI-TOF mass spectra: a systematic review. Clin Microbiol Infect 26:1310–1317.

4. Godmer A, Bigey L, Giai-Gianetto Q, Pierrat G, Mohammad N, Mougari F, Piarroux R, Veziris N, Aubry A. 2024. Contribution of machine learning for subspecies identification from Mycobacterium abscessus with MALDI-TOF MS in solid and liquid media. Microb Biotechnol 17.

5. Gibb S, Strimmer K. 2012. MALDIquant: a versatile R package for the analysis of mass spectrometry data. Bioinformatics 28:2270–2271.

6. Godmer A, Benzerara Y, Varon E, Veziris N, Druart K, Mozet R, Matondo M, Aubry A, Gianetto QG. 2023. MSclassifR: an R Package for Supervised Classification of Mass Spectra with Machine Learning Methods. bioRxiv 2022.03.14.484252.
